# Electronic properties of phosphorene nanoribbons with nanoholes†

**DOI:** 10.1039/c7ra12351e

**Published:** 2018-02-15

**Authors:** Lin Sun, Zhen Hua Zhang, Hao Wang, Mo Li

**Affiliations:** School of Physics and Electronics, Central South University Changsha 410083 China mo.li@gatech.edu +86 404 385 2472; School of Physics and Electronic Science, Changsha University of Science and Technology Changsha 410114 China; Hunan Province Higher Education Key Laboratory of Modeling and Monitoring on the Near-Earth Electromagnetic Environments (Changsha University of Science & Technology) Changsha 410114 China; Guangdong Provincial Key Laboratory of Micro/Nano Optomechatronics Engineering, College of Mechatronics and Control Engineering, Shenzhen University Shenzhen 518060 China whao@szu.edu.cn +86 0755 26557471 +86 0755 22673522; School of Materials Science and Engineering, Georgia Institute of Technology Atlanta, Georgia 30332 USA; State Key Laboratory for Powder Metallurgy, Central South University Changsha Hunan 410083 PR China

## Abstract

Using first-principles calculation based on density-functional theory, the electronic properties of monolayer black phosphorus nanoribbons (PNRs) with and without punched nanoholes (PNRPNHs) and their mechanical stability are studied systematically. We show that while the perfect PNRs and the PNRPNHs have similar properties as semiconductors in both armchair-edge PNR and zigzag-edge PNR structures, the nanoholes can lead to changes in the electronic structure: the zigzag-edge PNRPNH undergoes a direct-to-indirect bandgap transition while the armchair-edge PNRPNH still retains a direct bandgap but with a significant increase in the bandgap as compared to the perfect PNRs. We found also that nanoholes have little influence on the structural stability of PNRs; but the applied external transverse electric field and strain can be more effective in modulating the bandgaps in the PNRPNHs. These new findings show that PNRs are a promising candidate for future nanoelectronic and optoelectronic applications.

## Abstract 2

Using first-principles calculation based on density-functional theory, the electronic properties of monolayer black phosphorus nanoribbons (PNRs) with and without punched nanoholes (PNRPNHs) and their mechanical stability are studied systematically.

## Graphical abstract

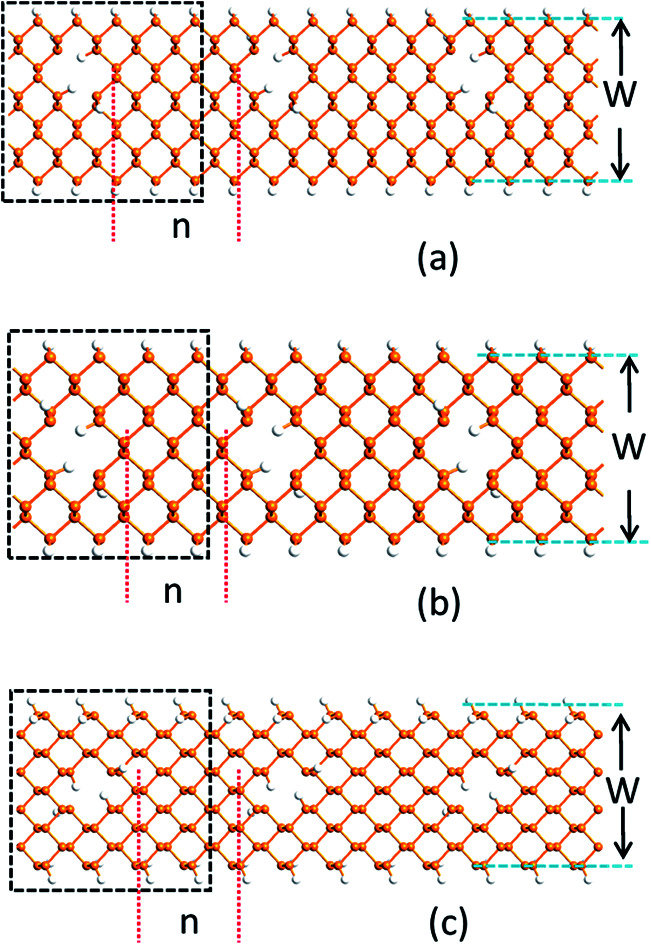

## 1. Introduction

With many remarkable properties such as high carrier mobility, the electronic properties of the 2D material graphene and its related devices have attracted tremendous attention.^1–7^ However, its application in the field of semiconductors is seriously limited by the absence of intrinsic bandgaps. Other 2D materials have been explored, including h-BN sheets, silicene and transition metal dichalcogenides. These layered materials have intriguing properties complementary to graphene.^8,9^ In particular, some of these new 2D materials do not suffer from this problem, one of which is phosphorene or monolayer black phosphorus fabricated using the same method as graphene.^10–18^ It is reported that phosphorene has a finite direct bandgap ranging from about 1.5 eV for a monolayer^19,20^ to 0.3 eV for multilayers,^21,22^ the drain current modulation up to 10^5^, and a room-temperature carrier mobility up to 1000 cm^2^ V^−1^ s^−1^, making it one of the most promising 2D materials in nanoelectronics applications, such as switch in transistor devices.

The electronic properties of phosphorene nanoribbons (PNRs) depend on the crystal orientation of the ribbons and boundary conditions. The PNRs and nanoflakes with different edge structures can be made if the phosphorene is cut along different directions. There are two typical types of PNRs, armchair-edge (APNRs) and zigzag-edge PNRs (ZPNRs). The pristine ZPNRs are metallic, independent of the ribbon width, while the pristine APNRs are semiconductors.^23^ In addition, surface adsorption and doping also can give rise to variety of changes in structural, electronic and magnetic properties. PNRs with the edge P atoms passivated by H atoms are direct-gap semiconductors but their band gaps are strongly dependent of the ribbon width resulted from quantum confinement effect.^24^ A study of the edge effects on the electronic properties of the PNRs with the edge functionalized with different chemical groups revealed that the APNRs are semiconductors for all edge groups, while ZPNRs are either semiconducting or metallic depending on different edge passivation.^25^

Besides these conventional ways in modifying the properties, the experiences learned from graphenes show us that there are other approaches that can also effectively change the electronic properties of 2D materials. It is found that by introducing certain types of “defects” or imperfections to graphenes, electronic properties can be modified, sometimes significantly. For example, punching nanoholes in 2D graphene plane, or artificially producing graphene nanomeshes (GNMs), can modulate the electronic structures effectively.^26–30^ The band gap is found to increase in the graphene with holes, making it a semiconductor out of semimetal.^31^ The zigzag-edge graphene nanoribbon with punched nanoholes (GNRPNHs) at the nonmagnetic state is always metallic regardless of neck widths, while the armchair-edge GNRPNHs have varying band gaps depending on the ribbon widths.^32^ While enlightening, these explorations so far have been limited only in graphenes. There is no similar effort reported for phosphorenes.

In this work, we present a systematic study of the effects of nanoholes on electrical properties in PNRs. In particular, we shall focus on periodic holes in the PNRs with different neck widths as a basic model before more complex holes and their geometries are explored. Through systematical control of the hole properties, we expect to see how they change the band structures and electronic transmissions, especially on relations between electronic properties and the neck width. In addition, we apply the external transverse electric field to examine how the perturbation changes the electronic properties. We found that the periodic nanoholes lead to transition from a direct-bandgap semiconductor into an indirect-bandgap one for the zigzag-edge PNRs, while for the armchair-edge PNRPNHs, the bandgaps significantly increase as the result of the presence of nanoholes. Another related issue in introducing holes is the structural stability for the “defected” PNRs. For this reason, we apply tensile strain to the PNRPNHs to investigate the structural stability and the resulting electronic property change as well.

## 2. Structure model and theoretical method

**Fig. 1:**
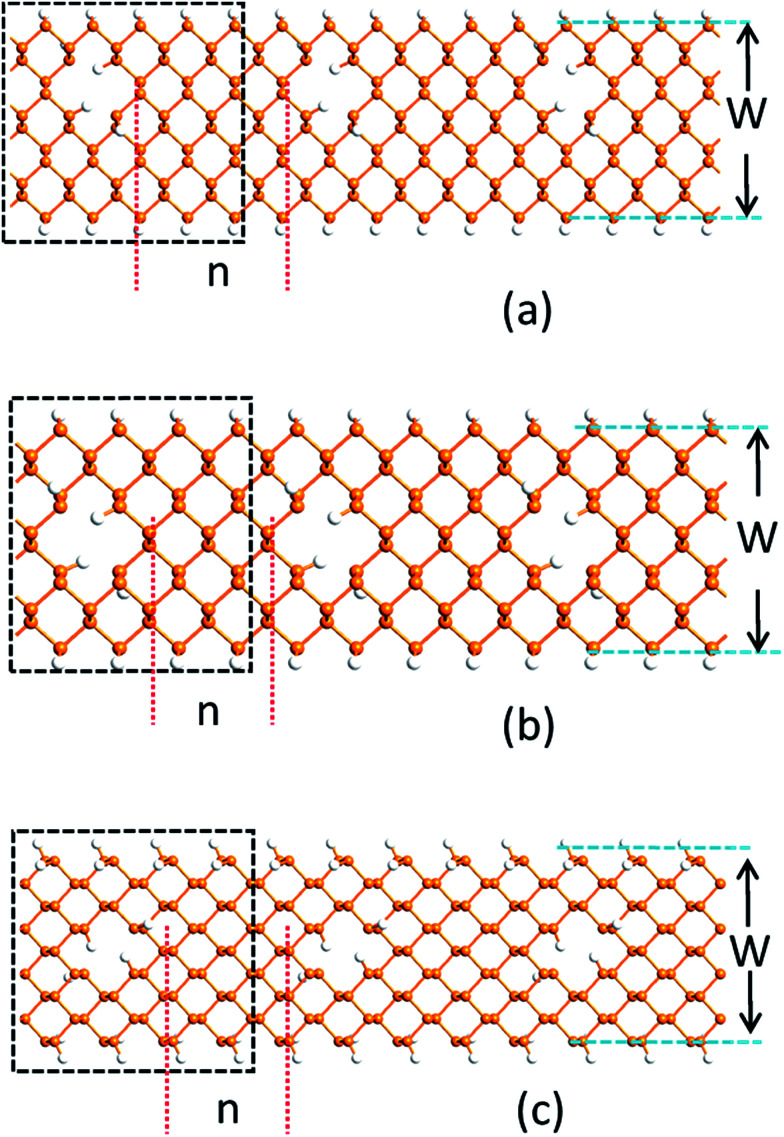
ZPNR (*n*, *W*)/APNR (*n*, *W*) superlattice models, where *n* is the neck width, and *W* the ribbon width, which is characterized by the number of phosphorus atom chains. The black dotted rectangle in figures exhibits a minimum repeated unit (supercell): (a) For ZPNR (4, 6) (E), (b) for ZPNR (3, 6) (M), (c) for APNR (8, 9). The orange and white spheres represent P and H atoms, respectively.

Fig. 1 shows the PNRPNH superlattice model used in this work, where *n* is the neck width defined as the minimum distance between two opposite edges of the adjacent nanoholes and characterized by the number of phosphorus atom chains. W is the ribbon width defined as the number of phosphorus atom chains along the ribbon width direction. The nanoholes are created by removing two adjacent P atoms periodically along the ribbon axis. For ZPNRs, *W* indicates the number of zigzag phosphorus chain, while for the APNRs, the number of phosphorus dimmer line. In the notation ZPNR (*n*, *W*), two different structures are considered: the first model marked as ZPNR (*n*, *W*) (M) describes the nanoholes punched in the middle (M) of the nanoribbons and symmetric with the nanoribbon axis, and the second, ZPNR (*n*, *W*) (E), has nanoholes near the upper edge of the nanoribbons. Fig. 1(a) and (b) show two examples, ZPNR (3, 6) (M) and ZPNR (4, 6) (E). Following the similar convention, APNR (6, 9) is shown in Fig. 1(c). A perfect ZPNR/APNR is denoted as ZPNR/APNR (*W*).

To stabilize the nanoribbon structures, hydrogen atoms are used to eliminate dangling bonds to passivate all the edges of both nanoholes and nanoribbons. As shown by the geometry optimization, sample size affects the results: the smaller the ribbon width characterized by *W*, the larger the effects caused by the distortion from the holes. In this work, we have *W* = 6 to 12 where the size effect is effectively reduced. The black dotted box in Fig. 1 exhibits a minimum repeated unit (supercell) which is used for calculation. As shown in Fig. 1, since the periodic boundary condition is used along the ribbon direction, an array of periodic holes appears in the PNRs although only one nanohole is present in the supercell.

The geometry optimizations and the subsequent calculations of electronic properties are performed by using Atomistix ToolKit (ATK),^33–37^ a first-principles method based on the density functional theory (DFT). The local density approximation (LDA) is used for the exchange correlation function. Troullier–Martins normconserving pseudopotentials are employed to represent the atom core; linear combinations of atomic orbitals are used for the valence states of electrons. The *k*-point sampling is chosen as 1 × 1 × 100 in the Brillouin zone and the cutoff energy is set at 150 Ry for the grid integration, mainly to control the size of the real space integral network partitioning and the solution of the Poisson equation. Since the phosphorene nanoribbon is one dimensional, we picked *k*-point meshes from 1 × 1 × 100 to 1 × 1 × 400 and for energy cutoff we had 100, 120, …, 160 Ry. The *k*-point and energy cut-off are tested and found sufficient to make all calculations converging well for the choice of 1 × 1 × 100 k-point mesh and energy cutoff at at 150 Ry. Full optimization of the atomic structures including the atomic positions and lattice parameters has been carried out with quasi-Newton method under the periodic boundary condition, and all calculations are performed after the geometry is optimized when all residual forces on each atom are smaller than 0.02 eV Å^−1^. The relaxed lattice constants for monolayer phosphorene are *a* = 3.262 Å and *b* = 4.476 Å which are in good agreement with experimental values^38^ and other theoretical calculations.^25^ The periodic boundary condition is used along the ribbon and vacuum layers of 15 Å both in plane and out of plane of the ribbons are used to avoid interaction between the periodic images.

## 3. Results and discussion

### 3.1 Electronic properties of the ZPNR with nanoholes

**Fig. 2:**
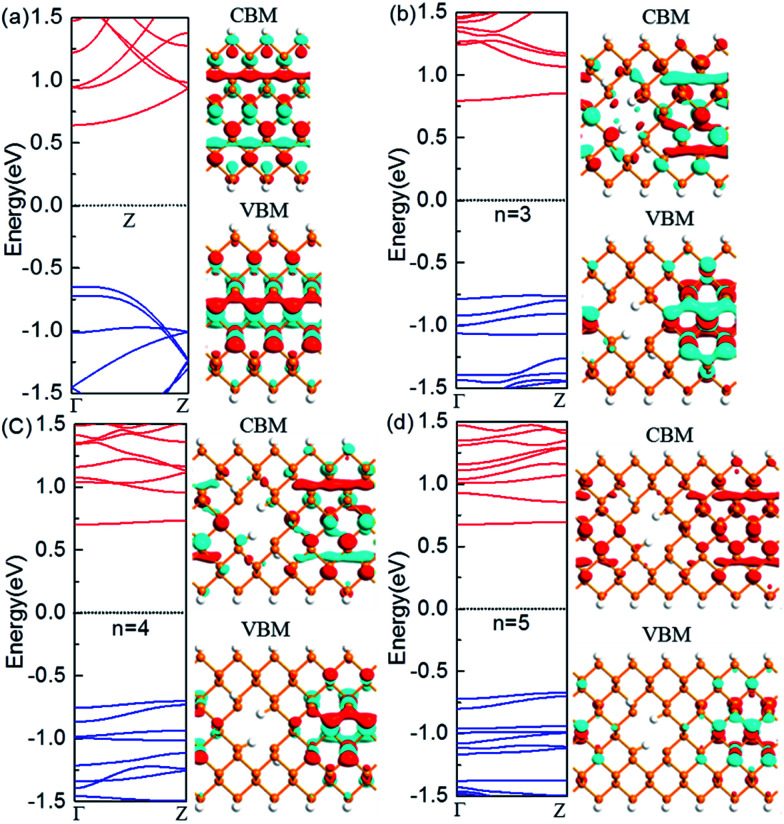
Computed band structures and Bloch states corresponding to the valence band maximum (VBM) and the conduction band minimum (CBM). The Fermi level is set to zero. (a) The perfect ZPNR (6), (b) ZPNR (3, 6) (M), (c) ZPNR (4, 6) (M) and (d) ZPNR (5, 6) (M). The orange and white spheres represent P and H atoms, respectively.

The nanoholes are characterized by the hole size and also neck width. Presently we only focus on the latter while leaving the former in the future work. To investigate the change of the electronic properties of the zigzag-edged PNRPNHs with nanoholes opened in the middle of the nanoribbons, we take the ZPNR (*n*, 6) (M) as an example. The band structures of the ZPNR (*n*, 6) (M) with the neck widths *n* = 3, 4, and 5 are shown in Fig. 2. For comparison, we draw the band structure of the perfect ZPNR (6) as marked by “Z”. Fig. 2(a) shows that ZPNR is always a semiconductor regardless of the size of the neck width. In other words, ZPNRs retain semiconductor properties even when holes are present as shown by the bandgap, the energy difference between the conduction band minimum (CBM) and valence band maximum (VBM). The perfect ZPNR has a direct bandgap of 1.29 eV, while the PNRPNHs have an indirect band gap. The band gap of the ZPNRs becomes wider after nanoholes are created, but narrows gradually with the increase of the neck width *n*. The semiconductor properties are enhanced at *n* = 3, 4.

Fig. 2 shows the Bloch states of the ZPNR (6) and ZPNR (*n*, 6) (M) (*n* = 3, 4, 5) corresponding to CBM and VBM. The results indicate that the nanohole can change the spatial distribution of wave function, or each atom's contribution to the quantum states. For the perfect ZPNRs, the wave function of the CBM is contributed by the central phosphorus atoms (see Fig. 2). In ZPNR, the wave function decays on two edges in CBM states, *i.e.*, there is little presence of the wave function distribution at the upper and lower edges of the nanoholes and most of the states concentrate in local area around the center between the nanoholes. As a comparison, the wave function of the perfect ZPNRs in VBM states is seen concentrated mainly in the middle of the ribbon. Therefore, the PNRs with periodic holes in the middle appear as made up of smaller nanoribbons between the nanoholes, the so-called neck-subprime nanoribbon (NSNR) and between the edge of a nanohole and the edge of a nanoribbon, the edge-subprime nanoribbon (ESNR). The NSNR is perpendicular to the direction of electronic transmission and the ESNR along the direction. The narrower ESNRs have stronger quantum confinement effects on the wave functions as seen in the diminishing edge states of the CBM. Consequently, the configurations of band structure vary as seen in the wider bandgap and smoother and flatter band structure along the wave vectors.

**Fig. 3:**
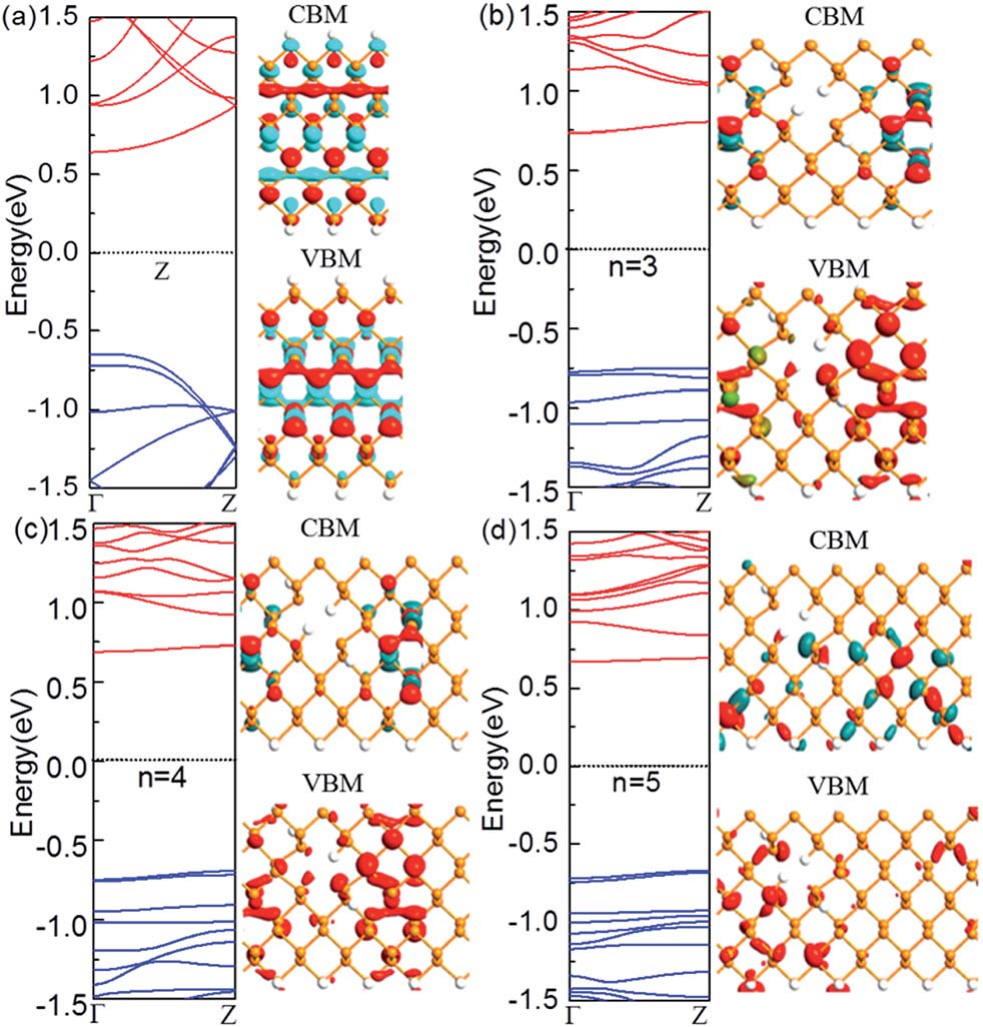
Computed band structures and Bloch states corresponding to the valence band maximum (VBM) and the conduction band minimum (CBM). The Fermi level is set to zero. (a) The perfect ZPNR (6), (b) ZPNR (3, 6) (E), (c) ZPNR (4, 6) (E) and (d) ZPNR (5, 6) (E). The orange and white spheres represent P and H atoms, respectively.

Next, we look at the influence of the location of the nanoholes in the ribbon. Fig. 3 shows the band structure of the ZPNR (*n*, 6) (E) with nanoholes near the upper edge of the nanoribbons with the neck widths *n* = 3, 4, and 5 respectively. The band structure of the perfect ZPNR (6) marked as “Z” is also plotted for comparison. From Fig. 2 and 3, we can see that band structures are different when nanoholes are at different locations. Among the most distinctive differences is the significant changes of the sub-band distributions in the energy space when the nanoholes are near the upper edge of the nanoribbons, while the variation of the bandgap is less obvious as compared with the case of nanoholes in the middle of the nanoribbons.

On the other hand, the corresponding Bloch states of the ZPNR (E) in the VBM and CBM states change significantly compared to the perfect ZPNR (6). As shown by Fig. 3, the Bloch states are not uniformly distributed. The wave functions are mostly centered around the phosphorus atoms on the NSNR and have little distribution around the nanoholes.

### 3.2 Electronic properties of the APNR with periodic nanoholes

**Fig. 4:**
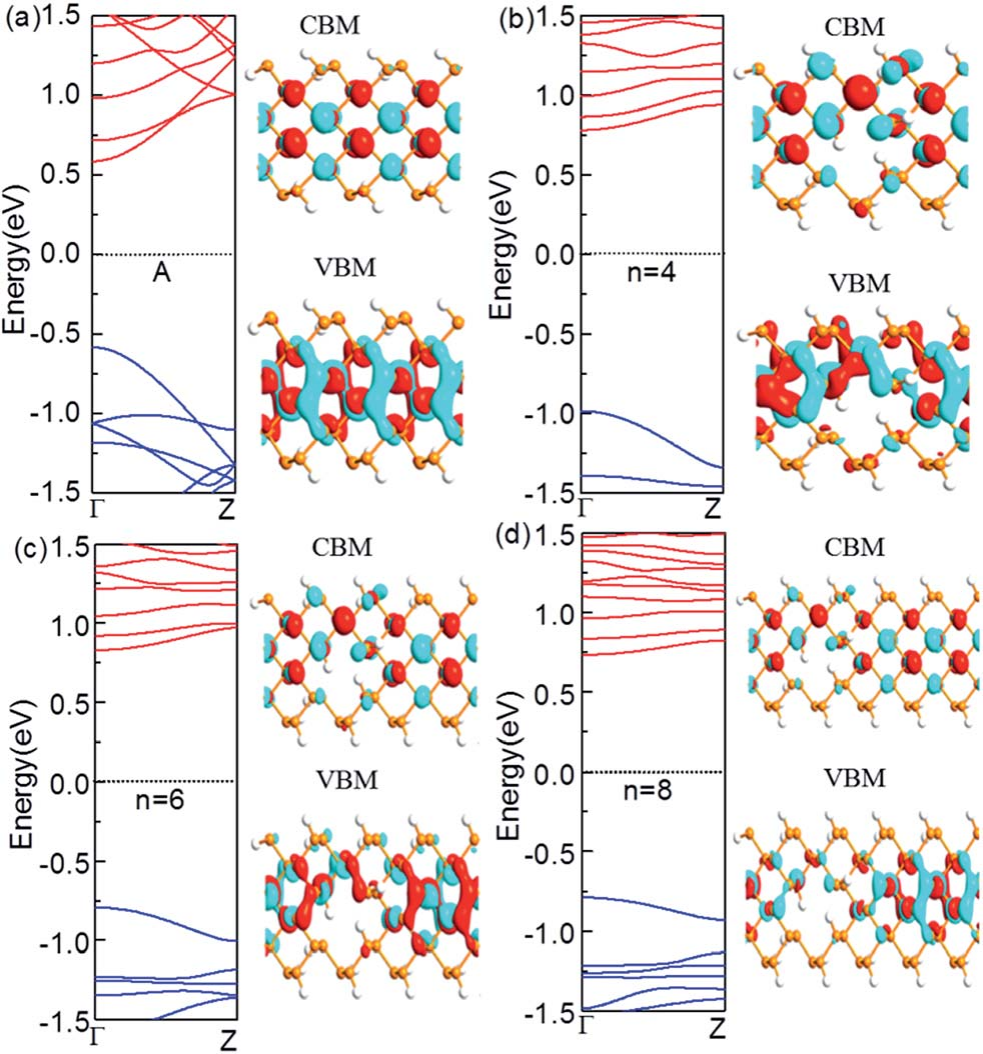
Computed band structures and Bloch states corresponding to the valence band maximum (VBM) and the conduction band minimum (CBM). The Fermi level is set to zero. (a) The perfect APNR (6), (b) APNR (4, 6), (c) APNR (6, 6) and (d) APNR (8, 6). The orange and white spheres represent P and H atoms, respectively.

For armchair-edged PNRPNHs, we chose APNR (*n*, 6) with the neck width *n* = 4, 6, and 8 respectively. The calculated band structures near the Fermi level are shown in Fig. 4. For comparison, we also plot the band structure of the perfect APNR (6) marked as “A”. The figure shows that the bandgaps of the punched ZPNRs increase significantly and become much larger than those of the perfect APNR (6) which is a semiconductor with a direct bang gap of 1.17 eV. When *n* = 4, the APNR still has a direct band gap which increases sharply to 1.77 eV. The reason is that when holes are formed in APNR (*n*, 6), the edge of the ESNR is still armchair shaped but its width decreases, which enhances the quantum-confinement effect that leads to a larger band gap. When the neck width increases, the ESNR plays a lesser role as the quantum-confinement effect weakens. Therefore, with increase of neck widths the band gap in general becomes narrower.

In comparison with those from the perfect APNRs, we plot the corresponding Bloch states of the APNRs in Fig. 4. One can see that the Bloch states have smaller amplitude and become more localized in the APNRs with holes that lead to the change of the band structure, especially the large band gap.

### 3.3 Density of states (DOS)

**Fig. 5:**
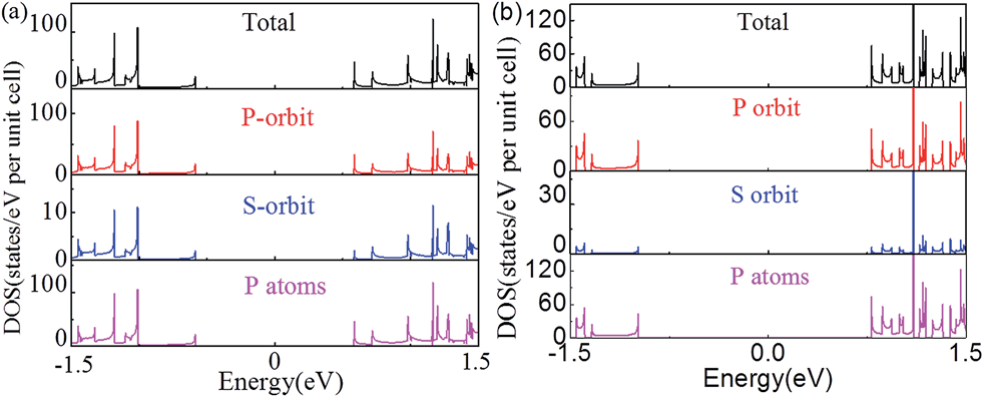
The total, p- and s-orbital, and P atoms projected density of states. (a) APNR6 and (b) APNR (4, 6). The Fermi level is aligned at zero.

The changes of the electronic band structure for the nanoribbons with nanoholes presented above are also reflected in the DOS. Fig. 5(a) and (b) present an example for the total DOS (TDOS) and projected DOS (PDOS) of the APNR (6) and APNR (4, 6). PDOS refers to the TDOS projected to different orbits and atoms, named as orbit-PDOS and atoms-PDOS, separately. The DOS of the perfect APNR in Fig. 5(a) suggests that the ribbon is a semiconductor, in which the TDOS is contributed mainly by the p-orbital and rarely by s-orbital.

For the APNRs with nanoholes, the position of VBM moves to the left while the position of CBM moves to the right. TDOS at VBM decreases gradually, even to zero at the energy between −0.5 and −1.0 eV. However, TDOS at CBM increases, suggesting that the nanoholes have changed the distribution of the DOS closing to Fermi level and reshaped the electronic band structure. From the perspective of orbit PDOS, P orbit still plays an important role in the conduction band and valence band. We should mention in passing that the DOS for ZPNRs are similar to APNRs'. To avoid repetition, we do not show the results here.

### 3.4 Mechanical stability of the nanoribbons with holes

When nanoholes are created, PNRs have the atomic configurations containing these defects. The next question is how these defects affect the relative mechanical stabilities of the PNRs. In other words, would the nanoribbons with holes relax locally, or even change to other structures? To this end, we calculate the binding energies for both zigzag- and armchair-PNRs with different neck widths. The binding energy *E*_B_ defined as *E*_B_ = (*E*_T_ − *n*_P_*E*_P_ − *n*_H_*E*_H_)/(*n*_P_ + *n*_H_) reflects the change of the structure stability of the sample. The formation energy of the nanoholes are show as the difference between *E*_B_ and the energy of the perfect PNRs, where *E*_P_ and *E*_H_ are the total energy of a single P and H atoms, respectively; *E*_T_ is the total energy of the PNRs with holes, and *n*_P_ and *n*_H_ are the number of P and H atoms in the PNR supercell, respectively.

Table 1 lists the binding energy of the ZPNR (*n*, 6) (M/E) *versus* the neck width, where ZPNR (M) and ZPNR (E) denote the nanoribbon with nanoholes in the middle and the upper edge. *n*→∞ corresponds to perfect ZPNR (6). As compared with the perfect PNRs, the formation energy shown in the parenthesis behind each *E*_B_ is slightly elevated, showing the defect structure indeed raises the total energy; but for different nanohole configurations they remain nearly the same for ZPNR (*n*, 6) (M) and ZPNR (*n*, 6) (E), indicating that the nanoholes of the size and location only affect the local atomic configurations, *i.e.* they do not result in structure change. Table 2 gives the binding and formation energies of the APNR (*n*, *W*) and APNR (*W*). In all cases, the PNRs with holes remain stable but with slightly elevated energy.

**Table 1:** The change of the binding energy of ZPNR (*n*, 6) (M) and ZPNR (*n*, 6) (E) *versus* the neck width *n*. The case of *n*→∞ corresponds to perfect ZPNR (6). The difference between the ZPNRs and the perfect PNR is shown in parenthesis, which indicates the relative difficulty in the nanohole formation. The unit is eV

Neck width	ZPNR (*n*, *W*) (M)	ZPNR (*n*, *W*) (E)
*n* = 3	−4.93 (0.05)	−4.93 (0.05)
*n* = 4	−4.98 (0.10)	−4.98 (0.10)
*n* = 5	−4.96 (0.08)	−4.96 (0.08)
*n*→∞	−5.08	−5.08

**Table 2:** The change of the binding energy of APNR (*n*, *W*) *versus* the neck width a, for simplicity, APNR (*n*, *W*) is written as APNR*W*. The case of a→∞ corresponds to perfect the APNR (*W*). The difference between the APNRs and the perfect PNR is shown in parenthesis, which indicates the relative difficulty in the nanohole formation. The unit is eV

Neck width	APNR6	APNR7	APNR8	APNR9	APNR10	APNR11	APNR12
*n* = 4	−4.65 (0.17)	−4.74 (0.15)	−4.81 (0.14)	−4.87 (0.13)	−4.82 (0.21)	−4.95 (0.12)	−4.99 (0.10)
*n* = 6	−4.62 (0.2)	−4.71 (0.18)	−4.78 (0.17)	−4.75 (0.25)	−4.89 (0.14)	−4.94 (0.13)	−4.97 (0.12))
*n* = 8	−4.72 (0.1)	−4.80 (0.09)	−4.86 (0.09)	−4.92 (0.08)	−4.96 (0.07)	−5.00 (0.07)	−5.03 (0.06)
*n*→∞	−4.82	−4.89	−4.95	−5.00	−5.03	−5.07	−5.09

### 3.5 The size quantum confinement effects on band gaps

**Fig. 6:**
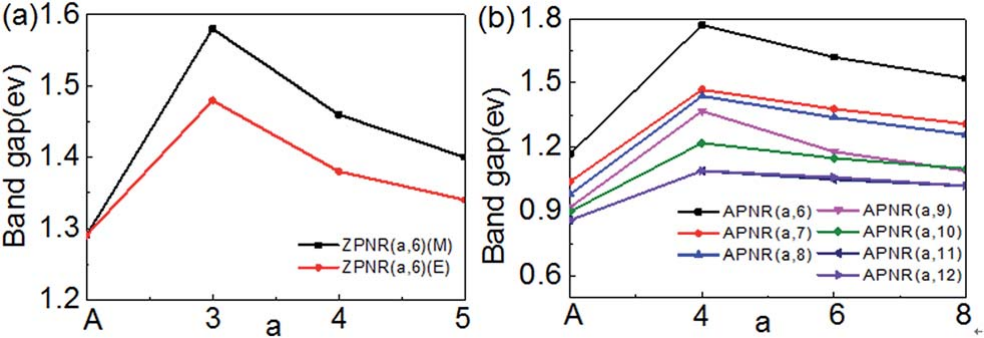
The band gap of the (a) ZPNR (n, 6), ZPNR (*n*, 8) and (b) APNR (*n*, *W*) as a function of ribbon width and the reciprocal of the neck width.

Next, we investigate how the PNR width and the neck width influence the electronic structures. Fig. 6 is the bandgap changes of the ZPNR (*n*, 6), ZPNR (*n*, 8) and APNR (*n*, *W*) *versus* the ribbon width and the reciprocal of the neck width. Two different representatives structures are examined, ZPNR (*n*, 6) (M), ZPNR (*n*, 6) (E), ZPNR (*n*, 8) (M) and ZPNR (*n*, 8) (E), respectively as shown in Fig. 6(a) (see Fig. S1 in the ESI† for detailed band structures).

Fig. 6(b) shows the bandgap of the APNR (*n*, *W*) with width *W* = 6 to 12. One can see that the bandgap decreases with increasing ribbon width due to ever weakening quantum confinement effect. The Bloch state of the ZPNR (*n*, 6) (M) shows that the wave function is almost entirely localized in the center. As a result, the bandgap of the ZPNR (*n*, 6) (M) is larger than that of the ZPNR (*n*, 6) (E) (Fig. 6(a)). The band gap of the perfect APNR is sensitive to changes of the bandwidth, and decreases monotonously with increasing ribbon width. In addition, when the bandwidth is bigger than 11, the band gap does not change much, still resembling that of monolayer phosphorene. The results are in good agreement with other calculations.^23^ Different from graphene nanoribbons which show an oscillatory behaviour as a function of ribbon width; monotonous width dependence of the band gap of PNRs makes PNRs a more promising candidate for PNRs-based FETs.

Note from the above discussion, the band gap of PNRs is determined by the intrinsic electronic states of phosphorus atoms. The scaling of the band gap with the ribbon width *d* obeys the usual 1/*W*^2^ relation according to quantum confinement.^24^ However, for both ZPNR and APNR, the band gap will increase obviously, when nanoholes are present. With the neck width increases, the band gap gradually decreases.

### 3.6 The effect of in-plane transverse electric field

**Fig. 7:**
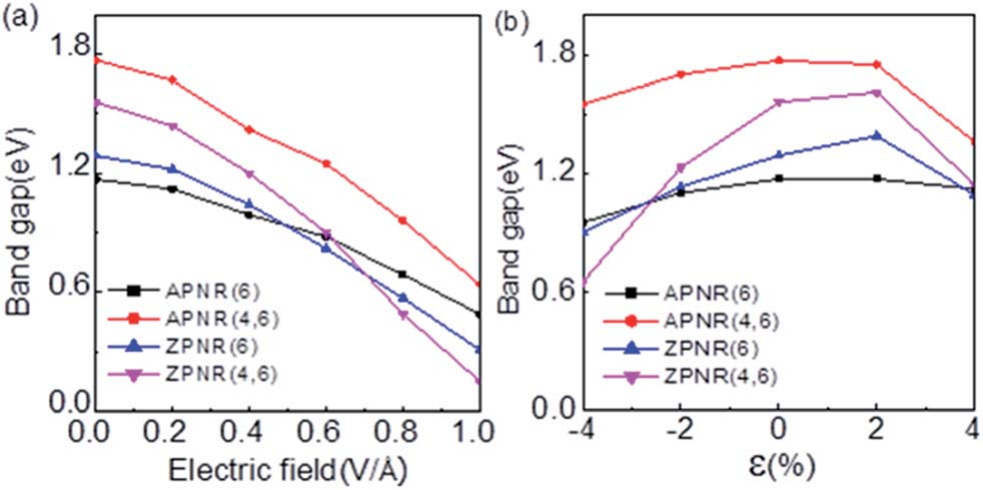
(a) Computed band gaps *versus* in-plane transverse electric field for the APNR (6), APNR (4, 6), ZPNR (6) and ZPNR(4, 6). (b) Computed band gaps of the APNR(6), APNR (4, 6), ZPNR(6) and ZPNR (4, 6) *versus* the tensile strain, ranging from −4% to 4%.Here, “ −” represents compression and “+” represents expansion.

Fig. 7 shows the band gaps of the ZPNR (*n*, 6) and APNR (*n*, 6) with the applied transverse electric field and tensile strain (see Fig. S2 in the ESI† for detailed band structures). As shown in Fig. 7(a), with the increasing transverse electric field, the band gaps of the PNRs decrease but with different rates. For perfect APNR with the bandgap at 1.17 eV, under in-plane transverse electric field of 1.0 V Å^−1^, the bandgap is reduced to 0.49 eV. For the APNR (4, 6), the bandgap initially at 1.77 eV, is reduced to 0.64 eV under the in-plane transverse electric field of 1.0 V Å^−1^.

**Fig. 8:**
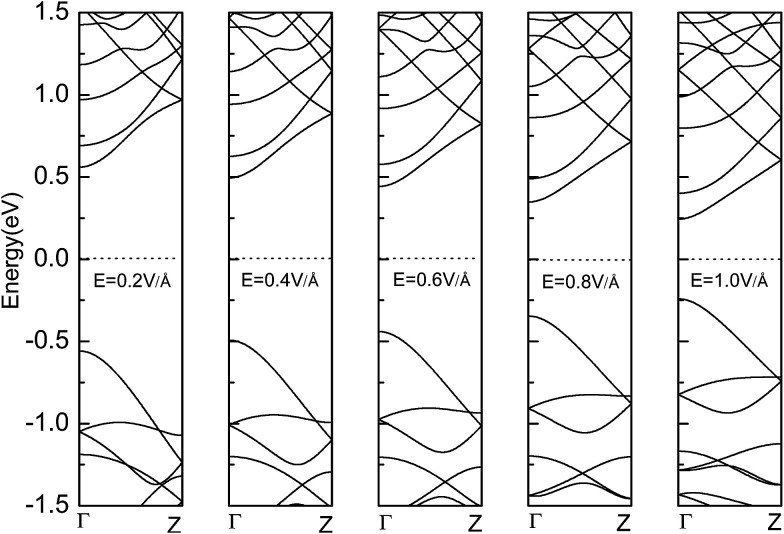
The band structure of APNR6 under applied electric field.

**Fig. 9:**
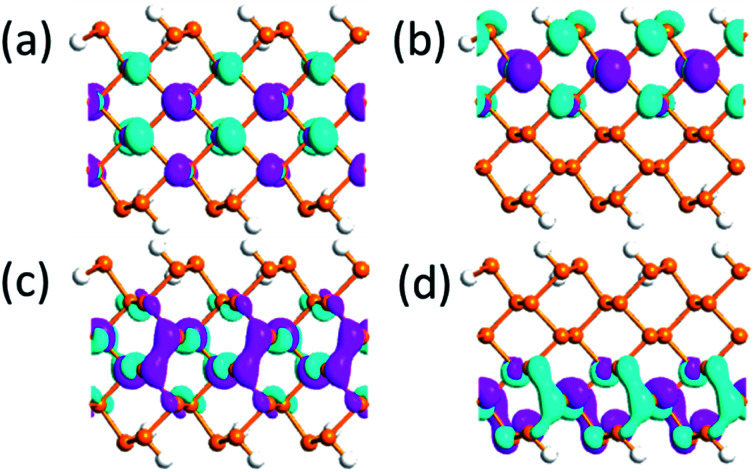
(a) and (b) The highest occupied and (c) and (d) lowest unoccupied molecular orbitals under 0 V Å^−1^ and 1.0 V Å^−1^ electric field, respectively, for the APNR(6) isovalue 0.03.

The bandgap reduction with increasing electric field is related to the charge redistribution and its induced dipole effects.^10,39,40^ As shown in Fig. 8, the band structure of APNR6 shows systematic change as the electric field increases. Both the conduction band and the valence band move toward the Fermi level and the band gap gradually decreases. As shown in Fig. 9, the highest and the lowest occupied molecular orbitals are polarized under the applied electrical field, *i.e.* they move in different directions toward the edges of the ribbon. The polarized charges creates dipoles that couple to the electrical field, leading to the decrease of the band energy.

The presence of the nanoholes reduces the bad gap more. For the ZPNR, without the transverse electric field, the band gap of the ZPNR (4, 6) is 1.56 eV but reduces to 0.15 eV with 1.0 V Å^−1^ transverse electric field, indicating that the ZPNR is more sensitive to transverse electric field. The stronger the transverse electric field, the faster the band gap drops. With further increase of transverse electric field, the band gap gradually approaches zero. These features are very desirable for development of nanoelectronic devices based on PNRs where the easy modulation of the electronic structure by transverse electric field is the basic requirement.

### 3.7 The effect of tensile and compressive strain

In addition, we investigated the effect of tensile strain on electronic properties of PNRs. As shown in Fig. 7(b) (see Fig. S3 in the ESI† for detailed band structures), the tensile and compressive strain *ε* is defined as *ε*= (*L* − *L*_0_)/*L*_0_, where *L* and *L*_0_ are the length of the stretch before and after the unit cell along the ribbon direction, respectively. For ZPNR/APNR (*n*, 6) as an example, we examined four cases, the perfect ZPNR/APNR with ribbon width *W* = 6, the ZPNR (3, 6) and APNR (4, 6). The results show that under compressive strain, from −4% to zero strain, the bandgap decreases. Under tensile strain, the bandgap increases first and at 2% tensile strain, the bandgap of the ZPNR further increases, but for APNR, the trend starts to reverse. At 4% tensile strain, the bandgaps of four cases decrease, among which the bandgap of the ZPNR (3, 6) has the largest change: rising from 0.65 eV at −4% to 1.61 eV at 2% strain but dropping to 1.14 eV at 4% strain. This result indicates that when nanoholes are present, the electronic structure becomes more sensitive to tensile strain. The bandgap of the APNR with holes decreases significantly at 4% tensile strain. The band structures corresponding to −4% and 4% strains are plotted in Fig. S3 in the ESI† where the band structure has transformed from the direct band gap into indirect band gap under tensile strain.

## 4. Conclusions

Using the first-principles method based on the density-functional theory, we investigated the electronic properties of monolayer black phosphorus nanoribbons with periodic nanoholes and their mechanical stability systematically. It is shown that the PNRs with nanoholes and the perfect PNRs have similar properties as semiconductors, regardless of their structures, *i.e.* armchair-edge PNRs or zigzag-edge PNRs.

However, the nanoholes can change the electronic structure of the PNRs and their responses to applied electrical field and strains. For example, the zigzag-edge PNRPNH is always semiconductor but undergoes a direct-to-indirect bandgap transition, and the armchair-edge PNRPNH remains a direct bandgap but the bandgap significantly increases as compared with the perfect PNRs. The influence of nanoholes on the mechanical stability of PNRs is primarily local structural relaxation around the holes. Moreover, as compared with the perfect PNRs, the applied external transverse electric field and strains can modulate the bandgaps of PNRPNHs more effectively. With the increasing transverse electric field, the band gaps of the PNRs decrease but with different rates, with further increase of transverse electric field, the band gap of the ZPNR (4, 6) gradually approaches zero. These novel electronic properties suggest that PNRs is a promising candidate for future nanoelectronic and optoelectronic applications. As in other 2D materials, phosphorus nanoribbons may possess other interesting properties which are currently under investigation.

## Conflicts of interest

There are no conflicts to declare.

## Supplementary Material

RA-008-C7RA12351E-s001
