# Supplementary material for: Electronic properties of phosphorene nanoribbons with nanoholes
Source: RSC Adv. 2018 Feb 15;8(14):7486–93. doi: 10.1039/c7ra12351e (PMC9078447; doi:10.1039/c7ra12351e)
Supplement: RA-008-C7RA12351E-s001 [file RA-008-C7RA12351E-s001.pdf]

Electronic Supplementary Material (ESI) for RSC Advances.
This journal is © The Royal Society of Chemistry 2018

Supporting Information:

S1

ZPNR6(M)

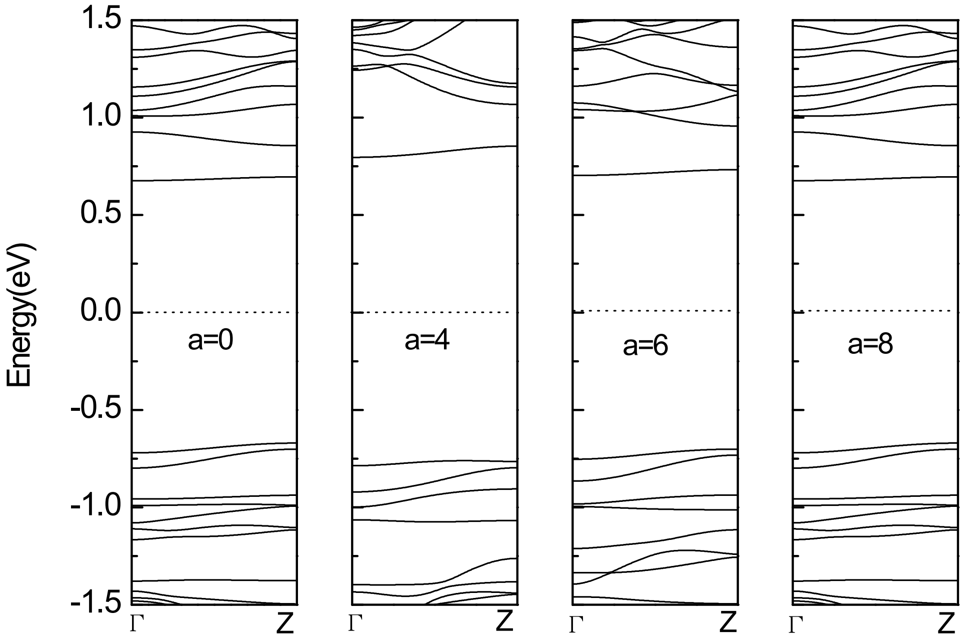

ZPNR6(E)

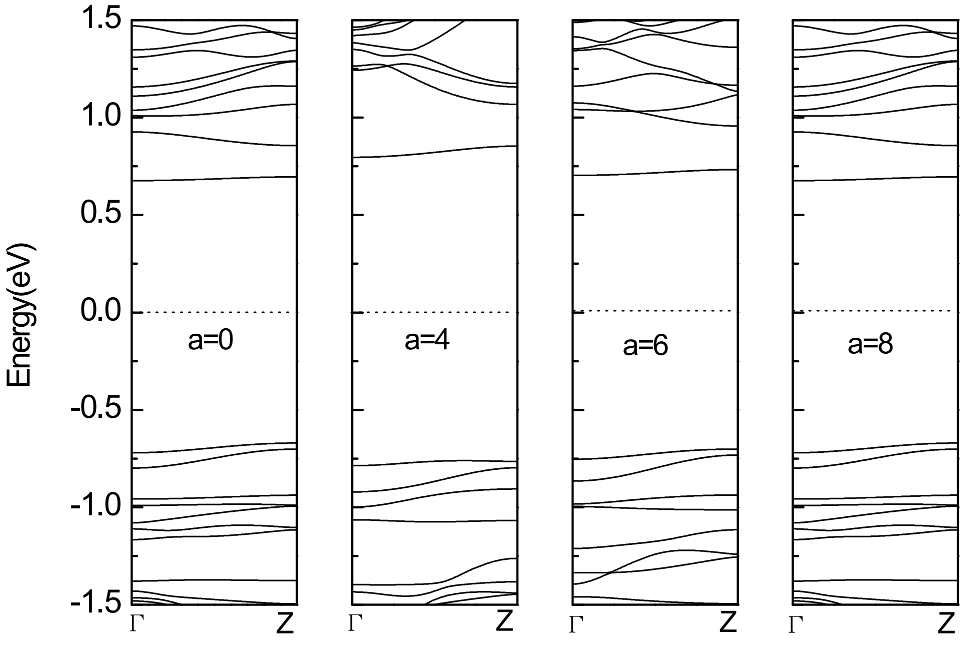

APNR6

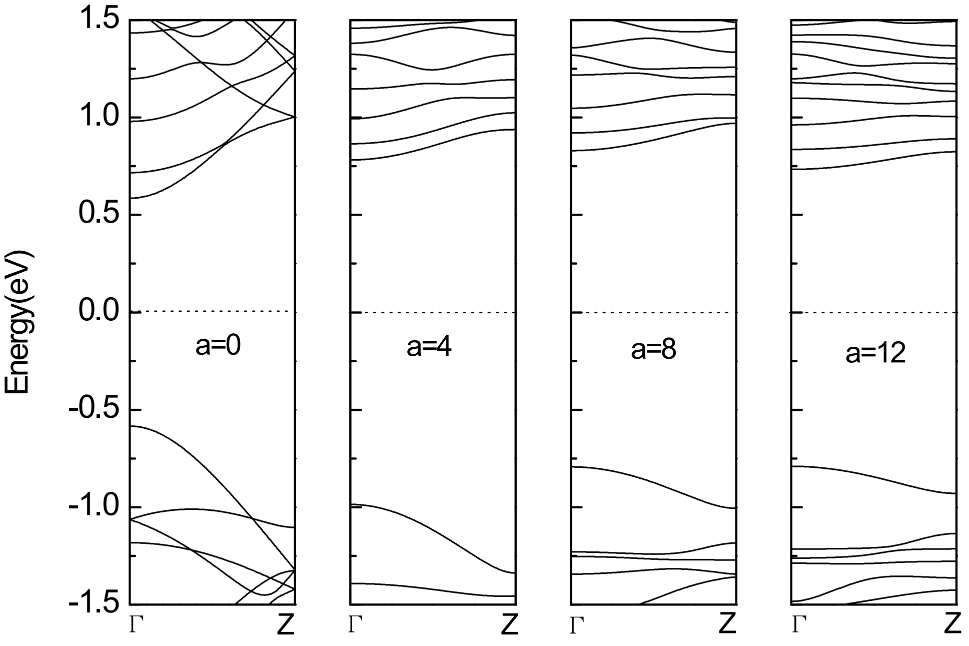

APNR7

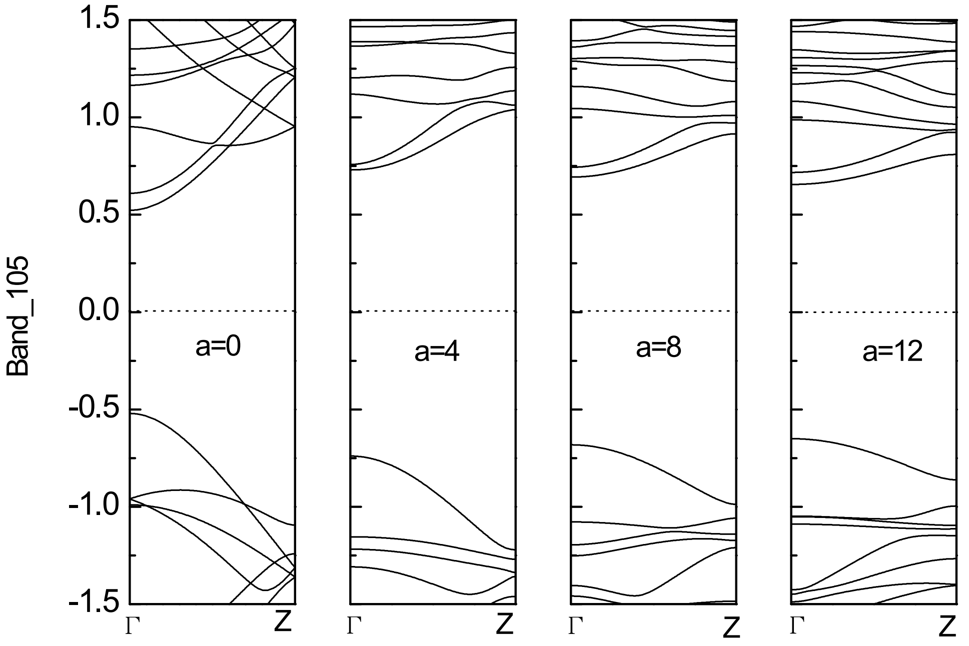

APNR8

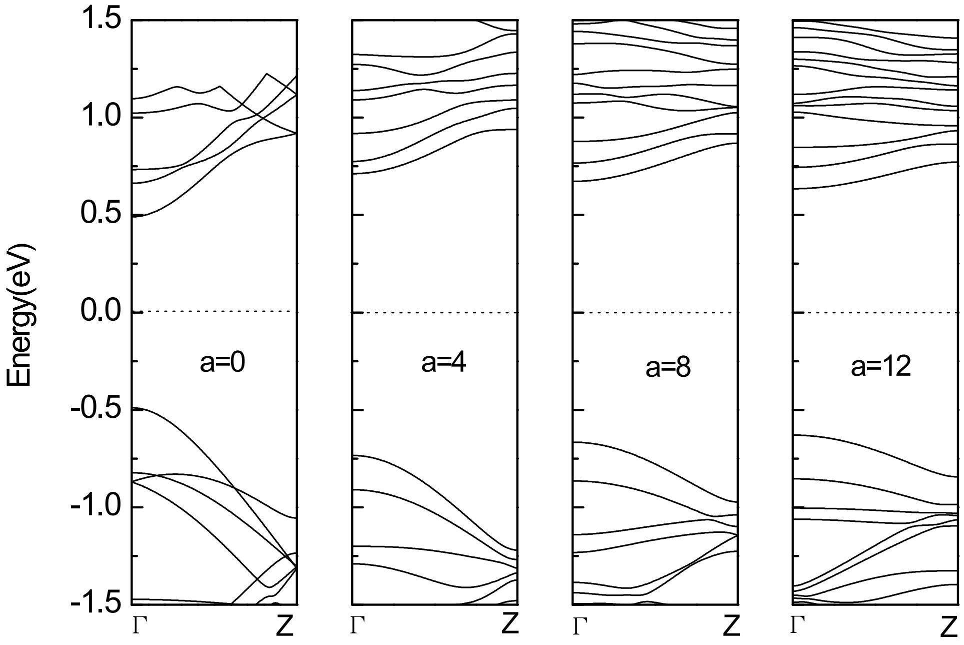

APNR9

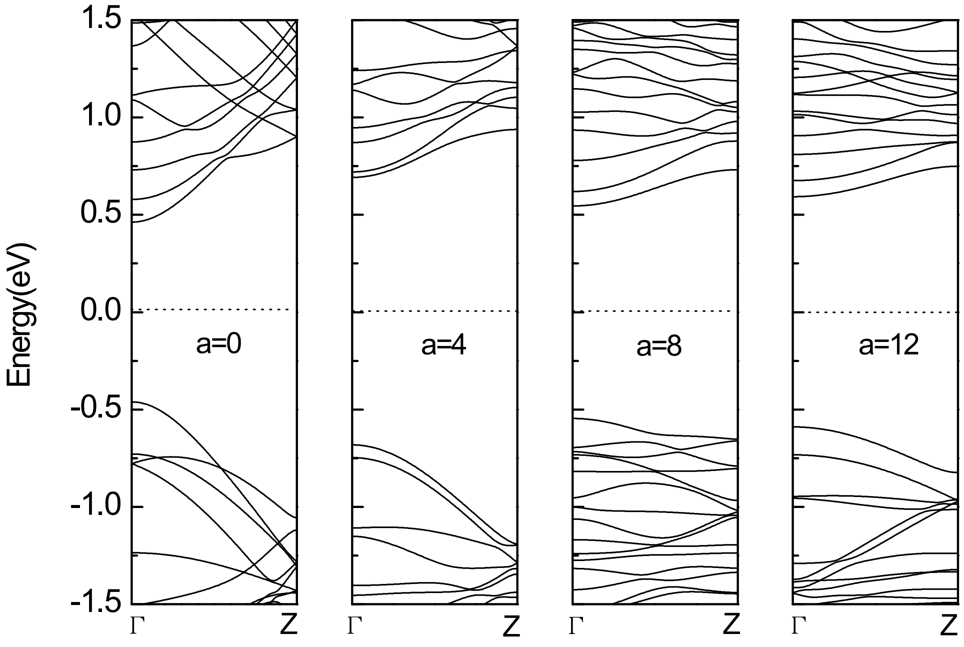

APNR10

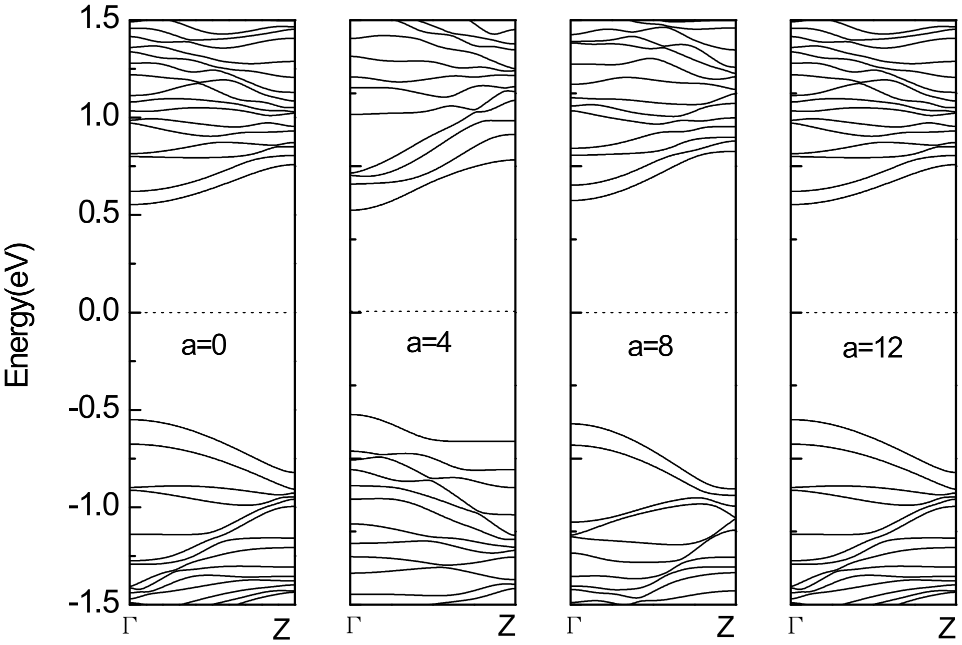

APNR11

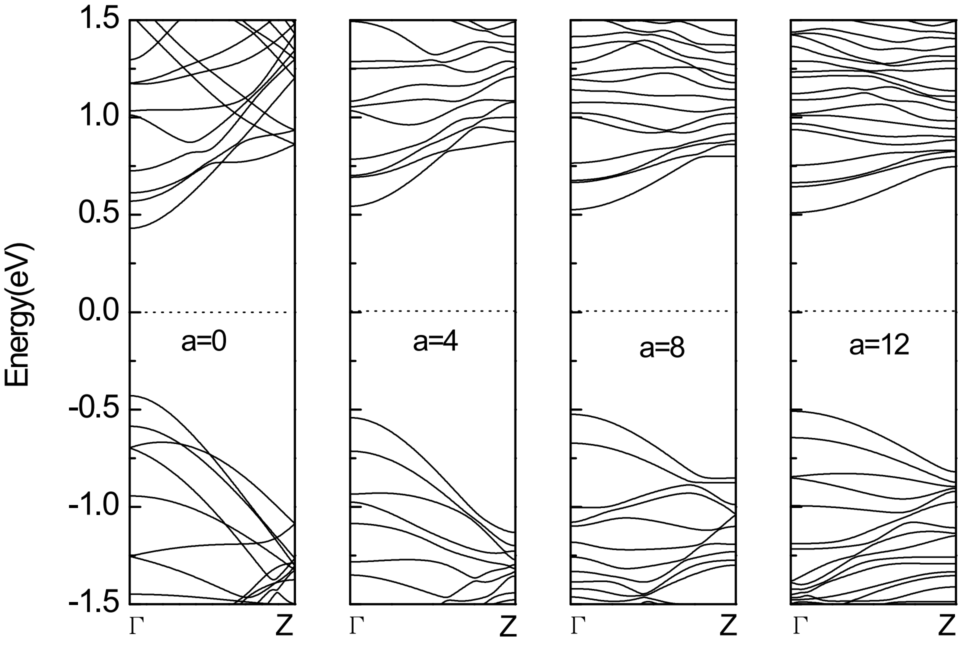

APNR12

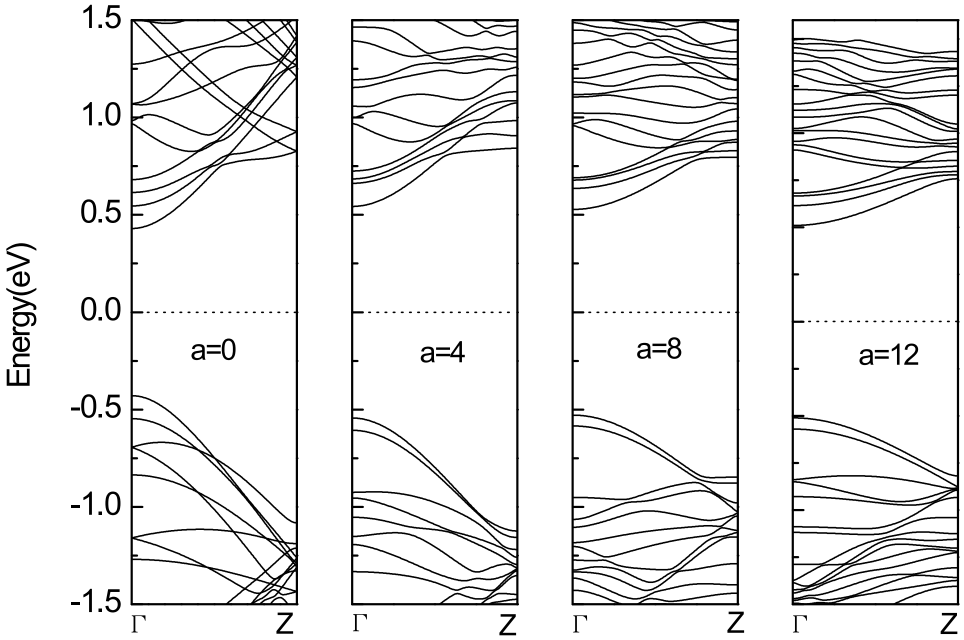

S2

APNR6

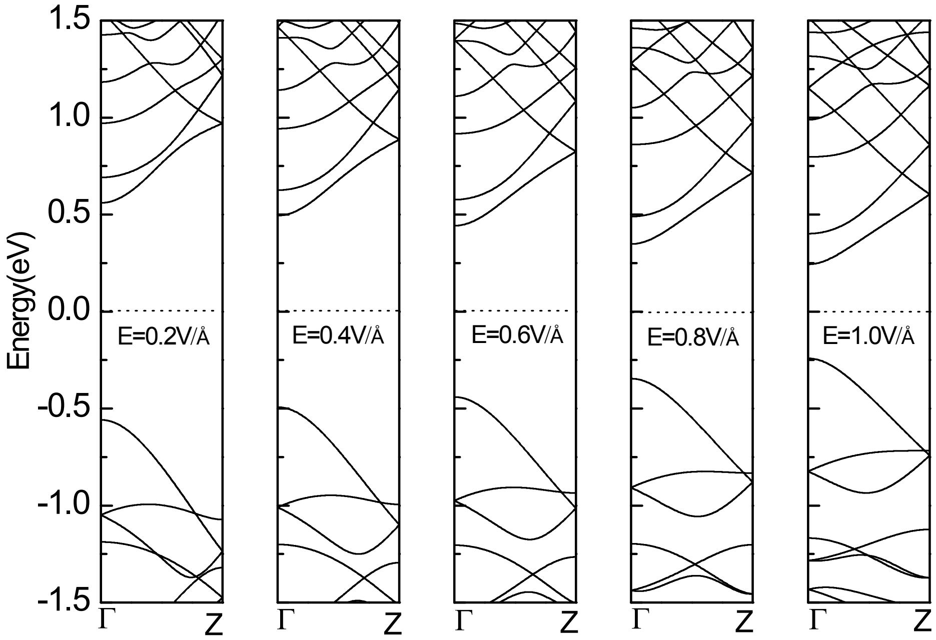

APNR（4，6）

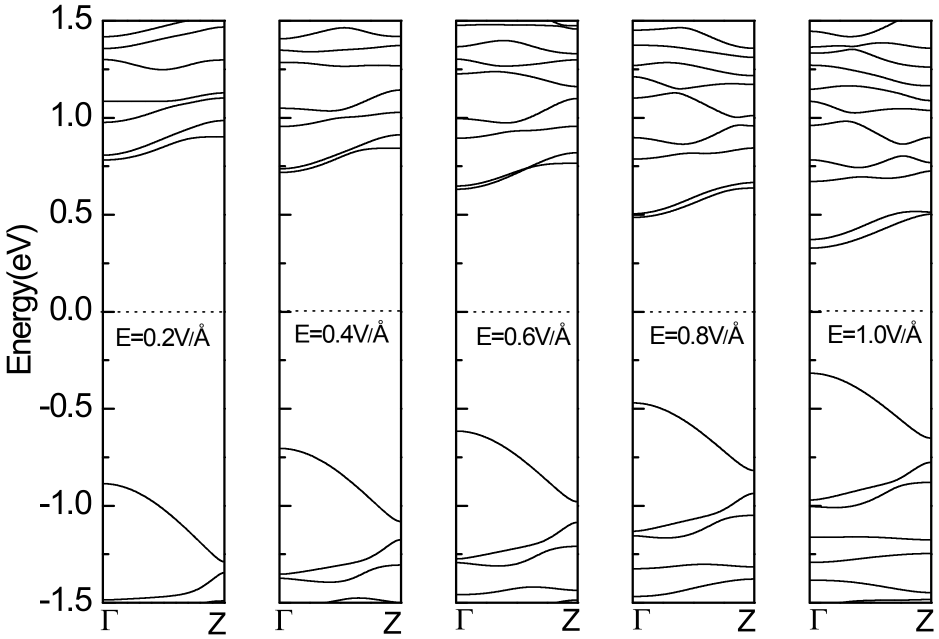

ZPNR6

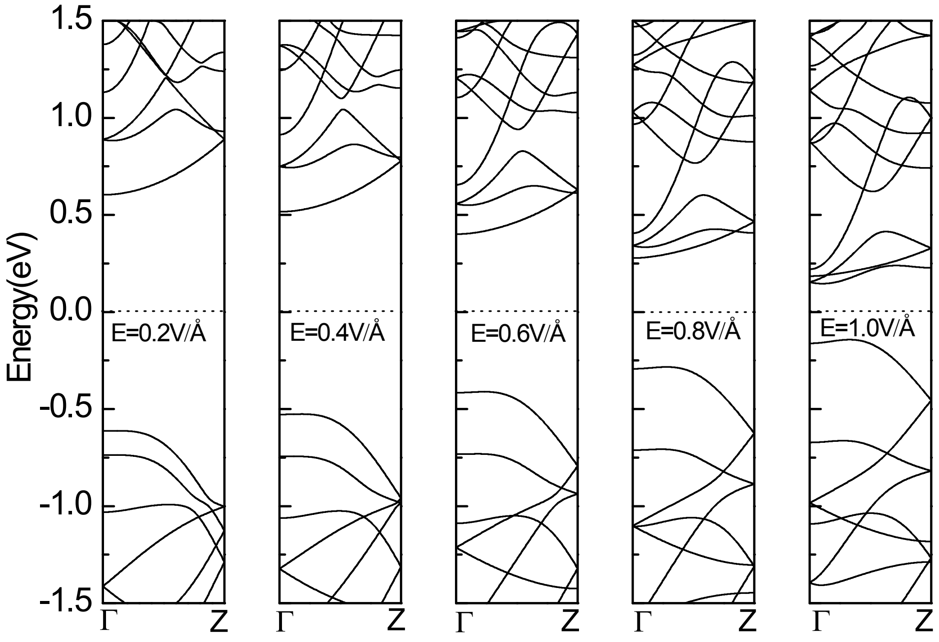

ZPNR（4，6）

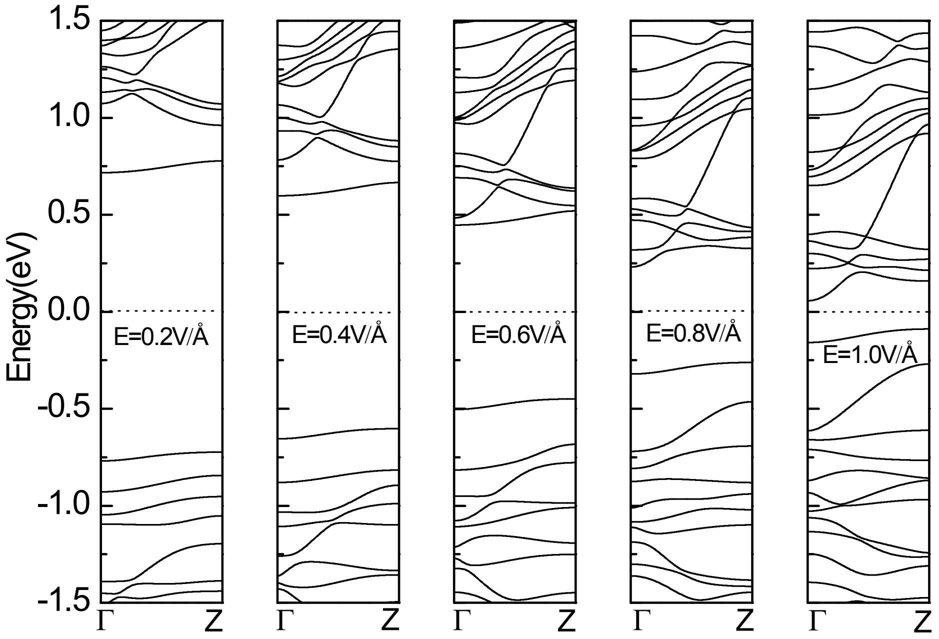

S3

APNR6

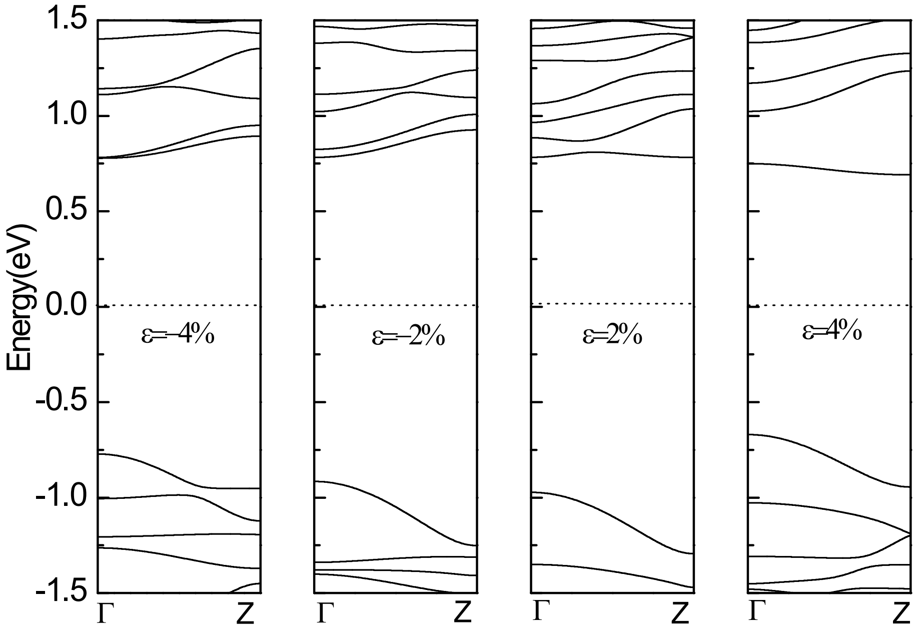

APNR(4，6)

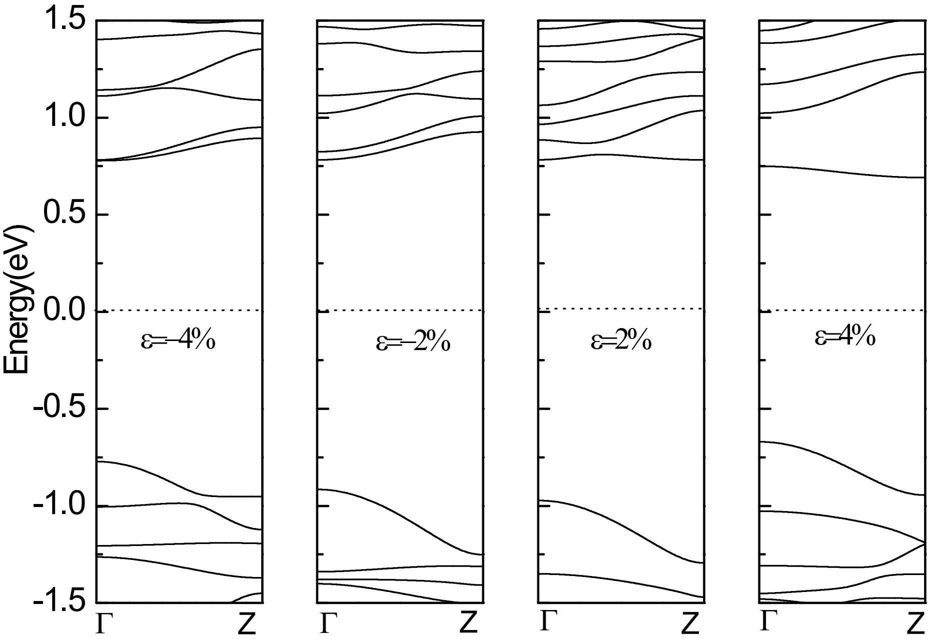

ZPNR6

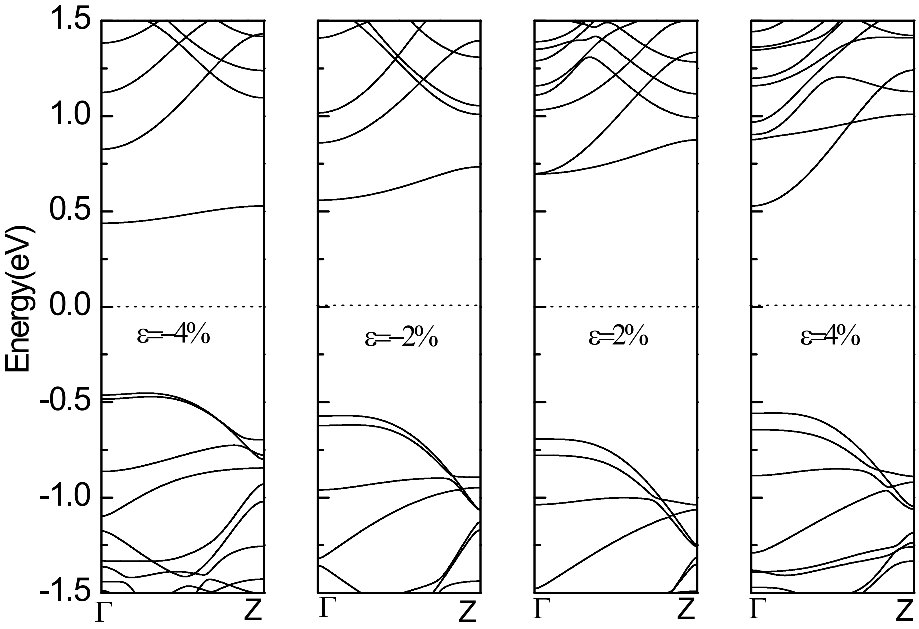

ZPNR(4，6)

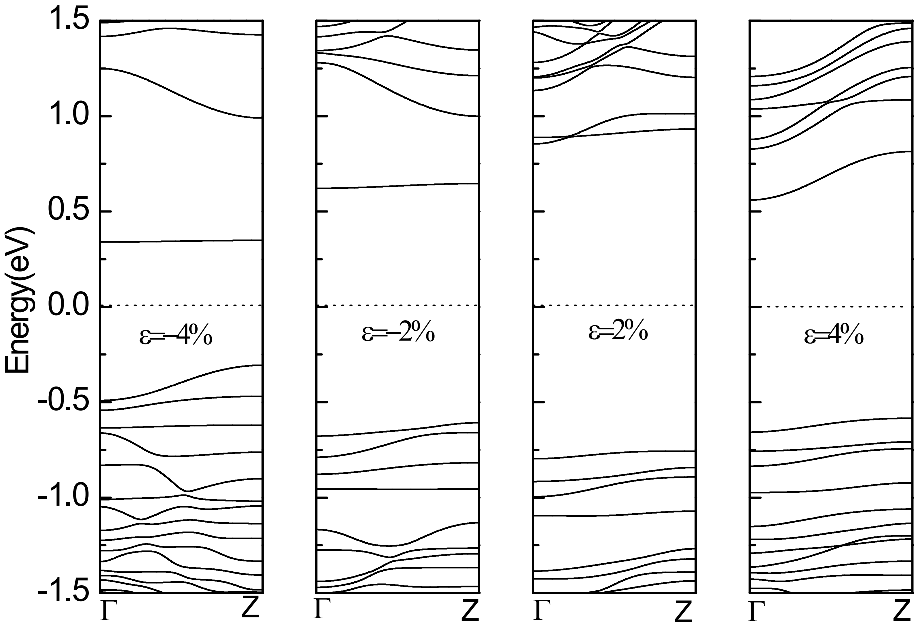
Energy(eV)
1.5
1.0
0.5
0.0
-0.5
-1.0
-1.5
ε=-4%
ε=-2%
ε=2%
ε=4%
Γ
Z
